# Biomarker-Guided Imaging and AI-Augmented Diagnosis of Degenerative Joint Disease

**DOI:** 10.3390/diagnostics15111418

**Published:** 2025-06-03

**Authors:** Rahul Kumar, Kyle Sporn, Aryan Borole, Akshay Khanna, Chirag Gowda, Phani Paladugu, Alex Ngo, Ram Jagadeesan, Nasif Zaman, Alireza Tavakkoli

**Affiliations:** 1 Department of Biochemistry and Molecular Biology, University of Miami Miller School of Medicine, 1011 NW 15th Street, Gautier Building, MC R629, Miami, FL 33136, USA; rxk641@miami.edu (R.K.); gowdachirag24@gmail.com (C.G.); axn668@med.miami.edu (A.N.); 2 Department of Medicine, Upstate Medical University Norton College of Medicine, Syracuse, NY 13202, USA; spornk@upstate.edu; 3 Department of Medicine, Robert Wood Johnson Medical School, New Brunswick, NJ 08901, USA; ab2584@rwjms.rutgers.edu; 4 Sidney Kimmel Medical College, Thomas Jefferson University, Philadelphia, PA 19107, USA; aya156@students.jefferson.edu (A.K.); phani.paladugu@students.jefferson.edu (P.P.); 5 Brigham and Women’s Hospital, Boston, MA 02115, USA; 6 Department of Computer Science, Whiting School of Engineering Johns Hopkins University, Baltimore, MD 21218, USA; ramjagad@cisco.com; 7 Cisco AI Systems, Cisco Inc., San Jose, CA 95134, USA; 8 Department of Computer Science, University of Nevada, Reno, NV 89512, USA; zaman@nevada.unr.edu

**Keywords:** osteoarthritis, joint pain, arthritis diagnosis, early arthritis detection, cartilage damage, joint inflammation, bone degeneration

## Abstract

Degenerative joint disease remains a leading cause of global disability, with early diagnosis posing a significant clinical challenge due to its gradual onset and symptom overlap with other musculoskeletal disorders. This review focuses on emerging diagnostic strategies by synthesizing evidence specifically from studies that integrate biochemical biomarkers, advanced imaging techniques, and machine learning models relevant to osteoarthritis. We evaluate the diagnostic utility of cartilage degradation markers (e.g., CTX-II, COMP), inflammatory cytokines (e.g., IL-1β, TNF-α), and synovial fluid microRNA profiles, and how they correlate with quantitative imaging readouts from T2-mapping MRI, ultrasound elastography, and dual-energy CT. Furthermore, we highlight recent developments in radiomics and AI-driven image interpretation to assess joint space narrowing, osteophyte formation, and subchondral bone changes with high fidelity. The integration of these datasets using multimodal learning approaches offers novel diagnostic phenotypes that stratify patients by disease stage and risk of progression. Finally, we explore the implementation of these tools in point-of-care diagnostics, including portable imaging devices and rapid biomarker assays, particularly in aging and underserved populations. By presenting a unified diagnostic pipeline, this article advances the future of early detection and personalized monitoring in joint degeneration.

## 1. Introduction

With its characteristic gradual cartilage degradation, subchondral bone remodeling, synovial inflammation, and finally joint failure, degenerative joint disease—especially osteoarthritis (OA)—represents a major burden on healthcare systems [1]. The pathophysiology consists of complicated interactions among mechanical, biochemical, and cellular processes that collectively cause articular cartilage degradation, development of osteophytes, subchondral bone sclerosis, and different degrees of synovitis [2,3,4]. Often, in cases where treatment procedures have limited effectiveness, traditional diagnostic techniques depend on radiography results that become evident only after significant tissue damage has occurred [5]. Moreover, the present diagnostic paradigm is not sensitive enough to identify early molecular and cellular changes that precede macroscopic tissue degradation, therefore delaying diagnosis and treatment during the most therapeutically sensitive period of illness [6,7].

From molecular pathways to tissue-level alterations, a systems medicine approach to degenerative joint disease diagnosis combines many data streams to provide a thorough evaluation of joint health across many biological levels [8]. By synthesizing information from circulating biomarkers, synovial fluid analysis, quantitative imaging parameters, and clinical evaluation, an integrated framework can help facilitate holistic orthopedic care and enable clinicians to detect disease earlier, stratify patients according to disease subtypes, predict progression trajectories, and monitor therapeutic responses with greater precision [9,10,11]. In this review, we provide a comprehensive analysis of current and emerging diagnostic tools for degenerative joint disease, with a particular focus on the integration of biochemical biomarkers, advanced imaging modalities, and artificial intelligence [12]. We begin by looking at the diagnostic value of cartilage degradation products, inflammatory mediators, and microRNA profiles—established and new molecular biomarkers [13,14]. We then review cutting-edge imaging modalities such as dual-energy CT, ultrasonic elastography, and T2 mapping MRI, stressing their particular benefits in spotting early joint abnormalities [15,16,17]. We subsequently discuss radiomics techniques, deep learning algorithms, and multimodal data integration to explore how AI can assist with image analysis and interpretation [18,19]. Through this comprehensive discussion, we aim to advance the understanding of biomarker-guided imaging and AI-enhanced diagnosis in degenerative joint disease, ultimately supporting the development of more sensitive, specific, and personalized diagnostic strategies [20] (Table 1). Accordingly, this review prioritizes diagnostic advancements directly supported by recent peer-reviewed studies and omits in-depth treatment or therapeutic discussions.

### 1.1. Methods

#### 1.1.1. Literature Search Strategy

A systematic literature search was performed across PubMed, Scopus, and Web of Science databases for studies published from 1 January 2013 to 15 March 2025. The search combined Medical Subject Headings (MeSH) and free-text terms related to (1) disease: “osteoarthritis”, “degenerative joint disease”, “cartilage degeneration”, “synovial inflammation”; (2) diagnostics: “biomarkers”, “MRI”, “ultrasound”, “dual-energy CT”, “radiomics”, “image analysis”; and (3) computational approaches: “artificial intelligence”, “machine learning”, “deep learning”, “multimodal integration”, and “clinical decision support”. Additional studies were identified through backward citation tracking and expert consultation.

#### 1.1.2. Inclusion and Exclusion Criteria

Included articles met the following criteria: (1) original studies or systematic reviews reporting diagnostic biomarkers or imaging findings relevant to osteoarthritis or degenerative joint disease; (2) studies that evaluated diagnostic accuracy (e.g., sensitivity, specificity, AUC), biomarker-imaging correlations, or AI-assisted image analysis; and (3) clinical studies or translational research involving human subjects. Exclusion criteria were (1) non-English language publications; (2) conference abstracts, editorials, or opinion pieces without primary data; (3) animal-only studies unless highly translational; and (4) articles focused solely on therapeutic interventions without diagnostic emphasis. Two reviewers independently screened titles and abstracts for eligibility. A full-text review was then conducted for shortlisted articles. Discrepancies were resolved by a third reviewer.

#### 1.1.3. Data Extraction and Synthesis

For eligible studies, the following data were extracted: Study design, sample size, diagnostic modality, type and source of biomarker or imaging data, statistical metrics of diagnostic performance, validation methods, and clinical integration outcomes. AI studies were additionally reviewed for algorithm type, feature extraction techniques, validation strategy, interpretability, and external generalizability. Given the heterogeneity in study designs and outcome metrics, findings were synthesized narratively and organized into thematic categories corresponding to biological scales (molecular, tissue, imaging, and computational integration). Key performance indicators and translational relevance were highlighted to inform practical clinical application.

## 2. Biochemical Biomarkers in Degenerative Joint Disease

### 2.1. Cartilage Degradation Markers

C-terminal cross-linked telopeptides of type II collagen (CTX-II) and cartilage oligomeric matrix protein (COMP) are among the many biochemical consequences of cartilage breakdown [21]. As diagnostic-prognostic biomarkers and quantifiable by enzyme-linked immunosorbent tests (ELISAs), they are particularly helpful [22]. Specifically, CTX-II is a fragment of type II collagen (90% of cartilage collagen) cleaved by collagenases, and is elevated in urine of knee OA patients, correlating with disease severity (higher in Kellgren-Lawrence grades 3–4 vs. 2) and MRI-detected structural abnormalities (osteophytes R = 0.330, bone marrow lesions R = 0.252, cartilage degradation R = 0.218, *p* < 0.05), with multivariate analysis highlighting osteophyte load and synovitis as predictors, suggesting contributions from bone and synovium [23,24,25].

COMP, a 524 kDa non-collagenous glycoprotein that can also be a beneficial biomarker [26]. Released into blood during cartilage turnover, it displays increased levels in OA patients, correlates with age, BMI, pain, and IL-1β, gender differences, and has negative associations with disease duration [27]. Though inter-laboratory standardization and age/sex/ethnicity-stratified reference ranges remain challenges for broad adoption, standardized early-morning urine sampling for CTX-II (normalized to creatinine), serum preference for COMP due to repeatability, and control of preanalytical variables (fasting, activity, handling)—with multiplex immunoassays and mass spectrometry enhancing sensitivity and throughput—have clear importance [28,29,30].

### 2.2. Inflammatory Cytokines and Mediators

Inflammatory cytokines play pivotal roles in the pathogenesis of degenerative joint disease, orchestrating catabolic processes that lead to progressive cartilage degradation, synovial inflammation, and bone remodeling [31,32,33]. As a key mediator in the inflammatory cascade, interleukin-1β (IL-1β) activates many downstream pathways that induce damage to joint tissues [34]. Multiple cell types, including synoviocytes, chondrocytes, and invading immune cells, synthesize IL-1β in osteoarthritic joints, thereby building a complex network of paracrine and autocrine signals [35]. Once bound to its receptor IL-1 RI, IL-1β begins signaling through the nuclear factor-κB (NF-κB) pathway [36].

IL-1β phosphorylates IκB kinase, then sets off the activation cascade by priming IκB proteins for ubiquitination and consequent proteasomal breakdown [37]. Released NF-κB first goes to the nucleus to regulate the transcription of numerous genes associated with inflammation, including those encoding matrix metalloproteinases (MMPs), cyclooxygenase-2 (COX-2), and inducible nitric oxide synthase (iNOS) [38,39]. Unquestionably, experimental studies show that IL-1β stimulation of chondrocytes greatly increases the synthesis of MMP-3 and MMP-13, which are enzymes required for the breakdown of type II collagen and proteoglycans, the main components of the extracellular matrix of cartilage [40]. By means of chondrocyte mortality, MMP activation, and proteoglycan synthesis inhibition, IL-1β induces iNOS expression, hence generating more nitric oxide (NO) and aggravating cartilage disintegration [41]. Because TNF-α signaling primarily via TNFR1 binds TRADD and RIPK1 to generate complexes that activate NF-κB, MAPKs, and caspases, IL-1β and TNF-α cooperate to enhance chronic inflammation in degenerative joint disease [42,43]. In those with osteoarthritis, increased TNF-α levels in their synovial fluid might match the degree of their symptoms and the radiographic change of their disease [44]. Comprising chondrocytes, mononucleated cells, and active synoviocytes, TNF-α generates autocrine loops that prolong inflammation while encouraging the manufacture of MMP and ADAMTs and thus impede the development of anabolic extracellular matrix [45]. By encouraging osteoclastogenesis and thereby reducing osteoblasts via both independent and RANKL-dependent mechanisms, it may also interfere with normal bone remodeling [46].

Through classical and trans-signaling involving IL-6R and gp130, IL-6 further amplifies this cytokine network, hence activating JAK-STAT pathways controlling inflammatory gene expression and cellular survival [47]. By MMP induction and RANKL/OPG axis control, IL-6 levels in OA fluid correlate with pain, synovial proliferation, and cartilage degradation indicators and control cartilage and bone metabolism [48,49]. These cytokines cumulatively reinforce inflammatory circuits, pushing OA development [50].

However, low circulating quantities and analytical restrictions of cytokine-based biomarkers make clinical use difficult [51]. Multiplex immunoassays permit more general profiling but suffer from cross-reactivity and matrix effects, whereas ELISAs offer sensitivity and specificity for particular cytokines [52]. Although it requires sophisticated procedures, mass spectrometry provides greater accuracy [53]. Further confusing metrics include preanalytical variability, including hemolysis, proteolysis, effusion dilution, and sample viscosity [54]. Critical is standardizing collection, processing, and storage; very important are contextual interpretation allowing for diurnal variance, concomitant inflammation, and cytokine-modulating treatments [55,56].

## 3. Advanced Imaging Modalities in Joint Disease Assessment

### Advanced Imaging Techniques for Degenerative Joint Disease

Advanced imaging modalities offer important new perspectives on the structural, compositional, and mechanical characteristics of joint tissues damaged by degenerative diseases, including osteoarthritis [57]. Among these, magnetic resonance imaging (MRI) methods—especially T2 mapping—have become absolutely essential for evaluating early degenerative changes and cartilage integrity [58]. By means of quantitative spin-spin relaxation times, T2 mapping provides an indirect evaluation of the collagen network and water content in cartilage [59]. Technical implementations produce parametric maps by means of multi-echo spin-echo sequences followed by signal decay curve fitting, so enabling pre-morphological cartilage degradation detection [60]. Clinically, high cartilage T2 values predict disease progression and match osteoarthritis risk factors [61]. Early compositional changes are enhanced by integration with other quantitative MRI techniques (e.g., T1rho, DTI, sodium imaging) [62]. While correlations with biochemical markers (e.g., CTX-II, COMP, IL-1β) confirm the potential of a multimodal assessment paradigm, reproducibility depends on standardizing acquisition parameters, fitting algorithms, and magnetic field strength [63,64,65].

Complementing MRI, ultrasonics, and elastography provide mechanical property assessment and real-time, radiation-free imaging [66]. With Doppler modes revealing inflammatory activity, conventional ultrasonic images show joint effusion, synovial hypertrophy, cartilage thinning, and osteophyte development [67]. By measuring tissue stiffness with strain or shear wave approaches, ultrasound elastography advances this [68]. High-resolution transducers and better beamforming methods are overcoming constraints, including cartilage thinness and joint accessibility, even if these issues still exist [69]. Good correlation between elastography and T2 mapping has been found to capture different but linked features of tissue degeneration [70]. Its dynamic properties during joint motion improve functional evaluations [71]. Comparisons with CT and integration with biochemical markers point to elastography’s important contribution in multimodal assessments [72,73]. When evaluating osseous structures and bone marrow changes, computed tomography (CT), especially using dual-energy technology (DECT), shines [74]. While conventional CT emphasizes subchondral sclerosis, osteophytes, and cysts, DECT uses virtual non-calcium imaging to enable the material breakdown and visualization of bone marrow edema-like signal intensity (ELMSI) [75]. Deep learning applications to CT and radiomics improve their possibilities for automated diagnostics even more [76]. Combining CT-based models with biomarkers and clinical data could create the backbone of thorough, individualized diagnostic systems [77]. These imaging modalities taken together support a systems medicine approach to joint disease: Bridging microstructural, mechanical, and molecular domains for early detection, prognostication, and therapy guidance [78].

## 4. Artificial Intelligence in Image Analysis and Interpretation

### 4.1. Machine Learning for Feature Extraction

Through image acquisition, preprocessing, region of interest (ROI) segmentation, feature extraction (first-order statistical, shape-based, second-order texture via GLCM/GLRLM, higher-order filtered features), feature selection, and model development, radiomics can essentially help transform imaging into high-dimensional data, capturing subtle patterns in joint structures like subchondral bone [79,80,81]. Using filter methods (correlation, mutual information), wrapper methods (recursive feature elimination, sequential selection), embedded methods (LASSO, elastic net, ridge regression), or dimensionality reduction (PCA, LDA, t-SNE) to maximize predictive performance and lower overfitting, feature selection helps to mitigate the curse of dimensionality [82]. Validation ensures reliability via internal methods (hold-out, k-fold cross-validation, bootstrapping) or robust external validation on independent multi-institutional datasets, employing discrimination (AUC-ROC, sensitivity, specificity), calibration (Hosmer–Lemeshow test), and clinical utility metrics (decision curve analysis), adhering to RQS and TRIPOD guidelines [83,84]. Technical challenges include image acquisition variability addressed by ComBat harmonization and intensity normalization, ROI segmentation variability tackled via semi-automated or deep learning-based methods validated against manual standards, and feature stability assessed through test-retest studies (ICC > 0.8) to ensure robustness against noise and protocol variations, with standardization via the Image Biomarker Standardization Initiative enhancing reproducibility and clinical translation [85,86].

### 4.2. Multi-Modal Data Integration

By combining multiple imaging modalities, clinicians can generate thorough joint assessments [87]. MRI excels in soft tissue visualization and compositional assessment; CT offers superior bone structure characterization; ultrasound provides dynamic, real-time evaluation; and nuclear medicine techniques, including positron emission tomography (PET), capture metabolic and molecular processes [88,89,90]. Beginning with spatial co-registration, which aligns pictures from several modalities to a single coordinate system, rigid (preserving distances between points) or non-rigid (allowing for local deformations) transformation techniques describe technological approaches to multimodal image integration [91]. Feature-level fusion combines retrieved features from separate modalities before analysis, using either simple concatenation or more advanced approaches such as canonical correlation analysis (CCA) or multiple kernel learning (MKL) to capture inter-modality correlations [92]. Decision-level fusion combines predictions or classifications from modality-specific models using voting systems, averaging, or meta-learner techniques that determine the best weighting based on individual model confidence [93]. Deep learning architectures designed for multimodal integration include dual-path networks that process each modality through specialized branches before fusion, cross-modal attention mechanisms that allow information to be exchanged between modality-specific features, and transformer-based approaches that capture complex inter-modality relationships via self-attention mechanisms [94,95]. Compared to single-mode evaluation, multimodal approaches combining T2 mapping MRI (assessing cartilage composition) with dual-energy CT (characterizing subchondral bone) have shown enhanced capability in applications of degenerative joint disease [96]. These technical methods allow a complete characterization of joint pathology spanning cartilage, bone, synovium, and supporting structures, thereby offering a more thorough evaluation of disease severity and progression [97].

Biomarker-imaging correlation models provide comprehensive perspectives on degenerative joint disease pathogenesis across biological dimensions by using quantitative connections between molecular markers and imaging characteristics [98]. Methodological techniques for developing these correlation models range from traditional statistical methods to cutting-edge machine learning methods [99]. Univariate correlation studies use Pearson’s or Spearman’s correlation coefficients to examine connections between individual biomarkers and imaging parameters, potentially revealing physiologically important associations [100]. Significant correlations have been seen, for example, between urine CTX-II levels and MRI-detected structural abnormalities such as osteophytes, bone marrow lesions, and cartilage degradation, suggesting that molecular cartilage degradation products reflect wider joint disease [23]. Multivariate regression models include many biomarkers and imaging parameters at the same time, assisting in the identification of independent correlations by addressing potential confounding variables [101]. Random forests, support vector machines, and neural networks, among other machine learning methods, capture complex non-linear correlations between molecular and imaging characteristics, potentially uncovering patterns that are unseen to classic statistical approaches [102]. Principal component analysis, t-distributed stochastic neighbor embedding, or uniform manifold approximation and projection can be used to visualize high-dimensional biomarker-imaging relationships and, potentially, identify patient subgroups with different molecular-imaging signatures through clustering and dimensionality reduction techniques [103]. Temporal correlation models with longitudinal data capture dynamic interactions between structural development and molecular changes, perhaps indicating early molecular alterations before significant imaging changes [104]. Implementation issues include standardizing biomarker measurement and image collection methodologies, accounting for biological variability in biomarker levels, and developing integrated databases including matching biomarker and imaging data [105]. Future prospects include the incorporation of spatially resolved molecular information using new technologies such as mass spectrometry imaging or targeted molecular imaging, allowing for direct spatial linkage between molecular processes and structural changes within joint tissues [106].

The incorporation of clinical data enhances diagnostic models by integrating patient-specific variables that influence illness appearance, progression, and treatment response [107]. Demographic characteristics (age, gender, ethnicity, body mass index) are clinically relevant to degenerative joint disease, as are symptom profiles (pain intensity, duration, pattern, functional limitations), physical examination findings (range of motion, crepitus, instability, alignment), comorbidities (diabetes, cardiovascular disease, osteoporosis), and treatment history (medications, physical therapy, injections, surgeries) [108,109,110]. Integration approaches range from simple rule-based algorithms to complex machine learning techniques [111]. Although this strategy may not completely capture complex relationships across data types, feature concatenation is a basic method for combining clinical variables with imaging and biomarker information prior to model building [112]. Ensemble approaches generate independent models for each data domain—clinical, imaging, and biomarker—and then combine their predictions using voting, averaging, or meta-learning methods to optimize weighting depending on individual model performance [113]. Multi-view learning approaches retain the unique statistical properties of each data domain while also capturing cross-domain interactions [114]. Multi-task learning uses shared representations across tasks to solve numerous related prediction tasks (e.g., diagnosis, prognosis, treatment response), improving overall performance [115]. A machine learning study for early-stage knee osteoarthritis used XGBoost to extract clinical features, convolutional neural networks for radiographic features, and demographic data to regularize intercorrelations among many features using L1-norm-based optimization [116]. This method performed better than single-modal strategies, highlighting the need for integrated evaluation [117]. Implementation issues include dealing with missing data, harmonizing different definitions across institutions, and developing interpretable models that enable clinical adoption [118]. Future prospects include the incorporation of new data modalities such as genetic information, physical activity monitoring, and patient-reported outcomes, potentially allowing for a more personalized evaluation of degenerative joint disease [119]. Despite promising results, AI applications in joint disease diagnosis face key limitations. Most current models rely on high-quality, annotated datasets that may not reflect real-world clinical variability, potentially limiting generalizability. Moreover, model performance can degrade in diverse populations due to bias in training data. The opacity of deep learning algorithms also challenges clinical adoption, as many models lack interpretable outputs that clinicians can trust. These limitations underscore the need for robust external validation, explainable AI frameworks, and regulatory oversight to ensure safety and equity in deployment.

## 5. Clinical Implementation and Validation

### 5.1. Diagnostic Performance Metrics

With threshold selection critically balancing these metrics based on clinical context and consequences of false positives versus false negatives, further complicated by the lack of strong reference standards like radiographic or arthroscopic evaluations, sensitivity and specificity analyses, fundamental to diagnostic test evaluation for degenerative joint disease, quantify the ability to identify affected individuals and healthy controls, respectively, and demand careful interpretation considering disease prevalence, spectrum bias, verification bias, and technical variability [120]. Receiver operating characteristic (ROC) curves comprehensively depict test performance across all threshold values, plotting sensitivity against 1-specificity, with the area under the curve (AUC) summarizing discriminative ability (0.7–0.8 acceptable, 0.8–0.9 excellent, >0.9 outstanding), enhanced by advanced methodologies like partial AUC, bootstrapped confidence intervals, and statistical curve comparisons, where decision thresholds are optimized using Youden’s index, diagnostic odds ratios, clinical utility, or cost-effectiveness analyses, enabling evidence-based implementation of biochemical biomarkers and imaging modalities [121,122]. Both heavily prevalence-dependent, thus requiring likelihood ratios and Bayesian frameworks for prevalence-independent interpretation, predictive values translate these metrics into clinically relevant probabilities [123]. Positive predictive value (PPV) indicates the likelihood of disease given a positive result, and negative predictive value (NPV) reflects the probability of no disease given a negative result, so guiding test deployment across varied settings from high-prevalence rheumatology clinics to low-prevalence screening [124]. The number needed to diagnose (NND) helps quantify testing efficiency [125]. Comparative effectiveness studies rigorously evaluate multiple diagnostic approaches, employing retrospective or prospective head-to-head designs, standardized protocols, and the GRADE framework to assess evidence quality, integrating bias risk, consistency, directness, and precision, demonstrating that quantitative MRI (e.g., T2 mapping) and ultrasound elastography offer complementary insights into cartilage composition and mechanics, while integrated multimodal algorithms enhance performance over single tests, with sequential testing optimizing resource use, ultimately informing tailored test selection, combination strategies, and resource allocation to improve diagnostic efficiency and accuracy in degenerative joint disease management across diverse clinical contexts [126,127,128].

### 5.2. Point-of-Care Applications

When combined with mobile health platforms and telemedicine, portable imaging technologies and fast biomarker assays are revolutionizing the way we diagnose degenerative joint disease by directly bringing point-of-care capabilities into primary care environments, rural clinics, and even community screening programs [129]. Although subtle cartilage lesions may still be a challenge, advances in miniaturized ultrasonicography (using 5–15 MHz broadband transducers, 20–30 Hz frame rates, synthetic aperture techniques, and wireless connectivity) and emerging low-field MRI systems (0.1–0.5 Tesla) equipped with permanent magnets, optimized pulse sequences, and AI-enhanced reconstruction have enabled surprisingly robust detection of joint effusion, synovial thickening, and osteophytes [130,131,132]. These technologies provide pragmatic, scalable answers when combined with organized training courses and flawless workflow integration [133]. Targeting inflammatory markers like IL-1β, TNF-α, and IL-6 as well as indicators of cartilage degradation, rapid biomarker assays—such as lateral flow tests using antibody-coated nanoparticles (yielding results in 10–20 min with nanogram-range sensitivity), microfluidic lab-on-a-chip platforms (employing colorimetric, fluorescent, or electrochemical readouts), and multiplexed biosensors with screen-printed electrodes and portable potentiostats—are being developed on the molecular front [134,135,136]. Together with implementation strategies targeted on user training, quality control, EHR integration, and regulatory compliance, successful clinical adoption depends on rigorous analytical validation (assessing sensitivity, specificity, precision, linearity, and interference) and demonstrating real-world utility via prospective studies [137]. Supported by cloud-based machine learning to customize illness monitoring, mobile health ecosystems further assist this diagnostic shift by including imaging, biomarker data, wearable motion sensors, and mobile applications for symptom tracking and rehab coaching [138]. Using remote assessments via live video, asynchronous messaging, or hybrid approaches, telemedicine ends the loop [139]. Particularly when combined with portable diagnostics and remote expert input, virtual care has demonstrated encouraging accuracy for spotting joint swelling and mobility restrictions using organized interviews, guided self-examinations, and smartphone-enabled evaluations [140]. More general acceptance still confronts challenges like digital infrastructure shortages, licensing restrictions, and varying degrees of reimbursement, however [141].

### 5.3. Clinical Workflow Integration

Implementing advanced diagnostic technologies for degenerative joint disease demands a multifaceted approach integrating implementation science frameworks like the Consolidated Framework for Implementation Research (CFIR), which delineates intervention characteristics (evidence strength, adaptability, complexity), outer setting (patient needs, external policies), inner setting (organizational culture, implementation readiness), individual characteristics (knowledge, self-efficacy), and implementation process (planning, execution, evaluation), necessitating comprehensive needs assessments, stakeholder engagement (clinicians, technologists, administrators, IT specialists, patients), and technical preparation (infrastructure, EHR/PACS/LIS integration, support mechanisms) [142,143,144]; workflow analysis via time-motion studies, process mapping, and discrete event simulation informs integration into clinical pathways, with implementation models ranging from disruptive redesign to incremental adoption, piloted in limited contexts to resolve challenges before scaling, monitored through metrics like utilization, diagnostic impact, and user satisfaction [145]. Physician training requires competency frameworks spanning theoretical knowledge (scientific principles, diagnostic performance), procedural skills (equipment operation, quality assessment), interpretive capabilities (pattern recognition, artifact identification), and professional attitudes (appropriate utilization, ongoing learning), delivered through didactic lectures, hands-on workshops, case-based learning, and simulation-based training (physical phantoms, virtual reality), with effectiveness assessed via knowledge tests, procedural checklists, interpretation metrics, and workplace observation, addressing barriers like time constraints and variable aptitude through protected training time, tiered education, super-user programs, and clear medicolegal guidelines [146,147,148]. Cost-effectiveness analyses employ cost-minimization, cost-effectiveness, cost-utility, or cost-benefit methodologies, evaluating direct (equipment, staff, consumables), indirect (patient time, productivity losses), and downstream costs (subsequent procedures, treatment modifications), using decision trees, Markov models, or discrete event simulation to project long-term outcomes, with sensitivity analyses ensuring robustness, as demonstrated by T2 mapping MRI and biochemical marker panels showing value through early intervention and patient stratification, informing targeted implementation, protocol optimization, and value-based reimbursement [149,150]. Regulatory pathways, such as FDA’s 510(k) clearance for Class II imaging devices or de novo/premarket approval for novel biomarkers, and EU MDR’s CE marking, mandate risk-based classification, analytical/clinical validation (accuracy, precision, sensitivity, specificity, clinical correlation), quality system compliance (ISO 13485; 2016—Medical Devices—Quality Management Systems—Requirements for Regulatory Purposes. International Organization for Standardization: Geneva, Switzerland, 2016) [151], and post-market surveillance, with emerging AI/ML frameworks addressing algorithm transparency and continuous learning, supported by early regulatory consultation, predicate device selection, and robust validation documentation, ensuring safety, effectiveness, and reliability in translating diagnostic innovations into clinical tools for degenerative joint disease management [152,153,154].

## 6. Future Directions and Emerging Technologies

### 6.1. Novel Biomarker Discovery

High-throughput screening and single-cell analytics have transformed biomarker discovery for degenerative joint disease by enabling comprehensive molecular profiling across genomic, transcriptomic, proteomic, and metabolomic domains, while longitudinal studies and standardization efforts enhance their clinical applicability [155]. High-throughput technologies, such as next-generation sequencing for genomic and transcriptomic profiling, mass spectrometry-based proteomics, and nuclear magnetic resonance for metabolomics, systematically evaluate thousands to millions of molecular candidates, identifying disease-relevant biomarkers with greater speed and specificity than traditional methods [156,157,158]. Single-cell RNA sequencing, ATAC-seq, proteomics, and spatial transcriptomics provide an unprecedented resolution of cellular heterogeneity, revealing distinct cell subpopulations and molecular signatures in joint tissues that drive pathogenesis, offering potential cellular biomarkers for precise disease classification and targeted therapies [159,160]. Longitudinal biomarker studies track molecular changes over time through repeated sampling, using advanced statistical models like mixed-effects modeling and trajectory analysis to capture dynamic patterns related to disease progression and treatment response, with findings indicating that biomarker rate of change often predicts outcomes better than static measurements [161]. Biomarker standardization addresses technical variability across preanalytical, analytical, and post-analytical phases, with international consortia like the FNIH and OARSI developing standardized protocols, reference materials, and quality management systems to ensure measurement reliability and facilitate clinical implementation [162]. Together, these approaches enable systems-level insights into disease mechanisms, improve prognostic stratification, and support the development of standardized, clinically actionable biomarkers for degenerative joint disease management, despite challenges like technical complexity, data integration, and evolving assay technologies [79].

### 6.2. Advanced Imaging Technologies

Molecular imaging techniques, such as positron emission tomography (PET), single-photon emission computed tomography (SPECT), optical imaging, and contrast-enhanced MRI, are revolutionizing degenerative joint disease assessment by visualizing specific biological processes at molecular and cellular levels, surpassing traditional anatomical imaging [163,164,165]. These methods employ molecular probes targeting biomarkers, receptors, or metabolic pathways, with PET using radiopharmaceuticals like 18F-fluorodeoxyglucose to quantify inflammation, 18F-sodium fluoride for bone remodeling, and others for specific joint degeneration processes [166]. SPECT offers cost-effective bone turnover assessment, while optical imaging excels in preclinical models but faces clinical translation challenges due to limited tissue penetration [167]. MRI with targeted nanoparticles combines excellent soft tissue contrast with molecular specificity [168]. Hybrid systems like PET/CT and PET/MRI integrate molecular and anatomical data, enhancing diagnostic precision, while emerging technologies like photoacoustic imaging and multiparametric MRI further expand capabilities [169,170]. Though it presents technological difficulties like field homogeneity and higher prices, ultra-high field MRI at 7 Tesla and above offers improved resolution and new contrast mechanisms, therefore permitting the early identification of cartilage and synovial changes [171]. Dynamic physiologically processes, including perfusion, tissue microstructure, and oxygenation, are captured by functional imaging, including blood oxygen level-dependent MRI, diffusion-weighted imaging, and dynamic contrast-enhanced MRI, so providing quantitative biomarkers for disease activity and therapeutic response [172]. Although they are mostly research tools, these advanced imaging techniques—which correlate molecular, functional, and anatomical data—promise earlier detection, improved patient stratification, and enhanced therapeutic monitoring, so guiding individualized treatment strategies for degenerative joint diseases despite obstacles including increased costs, technical complexity, and radiation exposure considerations [173].

### 6.3. Next-Generation AI Applications

Particularly for clinical and regulatory adoption, explainable artificial intelligence (XAI) techniques have become indispensable instruments in medical artificial intelligence systems to solve the intrinsic opacity of deep learning algorithms [174]. From interpretable models like decision trees and attention-based networks to post-hoc techniques like SHAP, LIME, and Grad-CAM, which provide explicit model evaluations in hindsight, these techniques span both. In joint imaging, for instance, attention mechanisms could emphasize radiographic features linked with osteoarthritis; SHAP or Grad-CAM representations let doctors identify which image regions or data points influence model predictions [175,176]. By tying internal model representations to clinically relevant events such as joint space decreasing or osteophyte growth, concept-based approaches like TCAV go one step further [177]. To guarantee that explanations remain practical without compromising technological viability or clinical throughput, these devices must strike a balance between interpretability, performance, and operating efficiency, however [178].

Through cooperative model training across institutions without exposing raw patient data, federated learning preserves privacy and increases model resilience, hence improving explainable artificial intelligence [179]. Because federated learning uses several datasets reflecting worldwide diversity in demographics, imaging modalities, and disease presentations, it is particularly helpful in osteoarthritis imaging [180]. It runs via distributed training and safe model change aggregation and is often complemented with methods such as homomorphic encryption or differential privacy [181]. Although technological difficulties arise from statistical heterogeneity and communication, asynchronous updates and local model customization might help to address these issues [182]. Increased generalizability, ongoing improvement from far-off data sources, and adherence to data security rules like HIPAA and GDPR are features of federated models [183]. Together with real-time analytics enabled by GPU/FPGAs, model compression, and edge computing, these developments form a transforming ecosystem in degenerative joint disease management that allows timely, individualized, clinically significant AI interventions [184] (Table 2).

## 7. Conclusions

In all, by integrating biochemical biomarkers, advanced imaging modalities, and artificial intelligence, clinicians can comprehensively approach degenerative joint disease diagnosis, enabling earlier detection, more precise characterization, and personalized monitoring of disease progression. While inflammatory cytokines, including IL-1β and TNF-α, reflect the important function of inflammation in disease pathogenesis, biochemical markers such as CTX-II and COMP offer molecular insights into cartilage degradation mechanisms. Complementing these molecular markers with new biomarkers like microRNAs and metabolomic signatures provides hope for identifying disease-related changes before structural changes show up on traditional imaging. Concurrently, advanced imaging technologies including T2 mapping MRI, ultrasonic elastography, and dual-energy CT enable the visualization and quantification of subtle tissue compositional changes, so providing spatial information that balances the systemic or local concentration measurements of biochemical indicators. By means of automated feature extraction, pattern recognition, and integration of several information sources, artificial intelligence applied to these intricate, multimodal datasets improves diagnostic capabilities, so possibly identifying disease signatures undetectable by conventional analysis.

The systems medicine approach presented in this review recognizes the multifactorial character of degenerative joint disease and the need for effective diagnosis depending on simultaneous assessment across several biological scales and pathophysiological processes. Integrated diagnostic systems provide a complete characterization of disease state and trajectory by including complementary information from molecular, cellular, tissue, and organ-level assessments rather than depending just on isolated biomarkers or imaging findings. This multiscale view fits the present knowledge of osteoarthritis as a whole-joint condition involving interrelated pathological processes across cartilage, bone, synovium, and supporting structures, rather than a simple cartilage disease. Moreover, the combination of several diagnostic modalities could help to solve the heterogeneity of degenerative joint diseases by allowing the identification of disease subtypes with different molecular mechanisms, structural expressions, and progression patterns that would profit from customized therapy approaches.

Bringing these cutting-edge diagnostic techniques into standard clinical practice still presents major difficulties. Widespread application depends on the technical standardization of both biomarker measurement and imaging acquisition, so guarantee result repeatability in many healthcare environments. Clinical validation studies have to show the incremental value of advanced diagnostics against traditional methods, proving significant changes in patient outcomes or management choices that support the extra complexity and resource use. Implementation plans have to take practical issues, including workflow integration, cost-effectiveness, accessibility, and training needs for healthcare providers, into consideration. Artificial intelligence algorithms could help to carefully negotiate regulatory paths for multimodal diagnostic approaches, including biomarker testing and imaging assessments, to ensure safety, efficacy, and appropriate clinical application.

The diagnosis of degenerative joint disease will be shaped in the future by increasingly tailored evaluations based on individual patient traits and particular clinical questions, together with standardized core measurements. Particularly for aging and underprivileged populations, point-of-care applications using portable imaging technologies, fast biomarker assays, and mobile health platforms potentially extend advanced diagnostic capabilities beyond specialized centers to primary care and community settings, so improving accessibility. While structured implementation science will enable efficient translation from research to clinical practice, continuous technological innovation, including molecular imaging approaches, ultra-high field MRI, explainable artificial intelligence methods, and federated learning frameworks, promises further refinement of diagnostic capabilities. Advancing biomarker-guided imaging and AI-augmented diagnosis of degenerative joint disease will help to provide early intervention, more accurate therapeutic targeting, and finally better outcomes for the millions of patients afflicted by these crippling diseases worldwide.

## Figures and Tables

**Table 1:** **Multilevel Diagnostic Framework for Degenerative Joint Disease.** This table outlines the systems medicine approach to diagnosing degenerative joint disease, particularly osteoarthritis. It captures the biological hierarchy from molecular mechanisms to tissue-level pathology, summarizing corresponding diagnostic tools across each level. The framework emphasizes integration of molecular biomarkers, quantitative imaging, and artificial intelligence to improve early detection, patient stratification, and longitudinal disease monitoring.

Biological Level	Representative Processes	Example Diagnostic Approaches
**Molecular**	Inflammatory signaling, matrix degradation	Biochemical biomarkers, synovial fluid analysis
**Cellular**	Chondrocyte apoptosis, immune infiltration	Single-cell analytics, histological methods
**Tissue**	Cartilage thinning, bone remodeling	Radiography, MRI, Ultrasound
**Functional/Clinical**	Pain, stiffness, mobility limitations	Clinical scoring systems, patient-reported outcomes
**Computational/Integrative**	Multimodal data fusion, risk modeling	AI-assisted interpretation, data integration tools

**Table 2:** **Integrated Technologies Advancing Degenerative Joint Disease (DJD) Diagnosis**. This table summarizes the multidisciplinary innovations underpinning modern DJD diagnostics. It highlights how high-throughput biomarker discovery, advanced imaging modalities, and explainable AI approaches collectively enhance disease characterization, prognostic stratification, and clinical decision-making. Each technological category is linked to its core platforms and corresponding clinical impact, emphasizing systems-level integration and translational potential.

Category	Key Technologies	Clinical Impact
**High-Throughput Biomarker Discovery**	NGS, Mass Spectrometry, NMR	Accelerates discovery of molecular signatures
**Single-Cell and Spatial Analytics**	scRNA-seq, ATAC-seq, Proteomics, Spatial Transcriptomics	Reveals cellular heterogeneity in joint tissues
**Longitudinal Biomarker Studies**	Mixed-effects modeling, Trajectory analysis	Tracks biomarker dynamics for prognosis
**Biomarker Standardization**	FNIH, OARSI protocols, Reference standards	Ensures reproducibility and clinical adoption
**Molecular Imaging (PET, SPECT, Optical)**	18F-FDG PET, 18F-NaF PET, SPECT	Visualizes inflammation and bone remodeling
**Advanced MRI Techniques**	7T MRI, BOLD-MRI, DCE-MRI, DWI	Quantifies disease activity at high resolution
**Hybrid Imaging Systems**	PET/CT, PET/MRI	Improves diagnostic precision via multimodal data
**Explainable AI (XAI)**	SHAP, LIME, Grad-CAM, TCAV	Enables transparent AI-driven decision making
**Federated Learning**	Federated training, Differential Privacy	Improves generalizability and data privacy
**Edge Computing and Model Compression**	Edge AI, GPU/FPGAs, Model compression	Supports real-time, low-latency diagnostics

## Data Availability

No new data were created or analyzed in this study. Data sharing is not applicable to this article.

## References

[1] Hunter D.J., Bierma-Zeinstra S. (2019). Osteoarthritis. Lancet.

[2] Loeser R.F., Goldring S.R., Scanzello C.R., Goldring M.B. (2012). Osteoarthritis: A Disease of the Joint as an Organ. Arthritis Rheum..

[3] Martel-Pelletier J., Barr A.J., Cicuttini F.M., Conaghan P.G., Cooper C., Goldring M.B., Goldring S.R., Jones G., Teichtahl A.J., Pelletier J.P. (2016). Osteoarthritis. Nat. Rev. Dis. Primers.

[4] Bijlsma J.W., Berenbaum F., Lafeber F.P. (2011). Osteoarthritis: An Update with Relevance for Clinical Practice. Lancet.

[5] Guermazi A., Hunter D.J., Roemer F.W. (2009). Plain Radiography and Magnetic Resonance Imaging Diagnostics in Osteoarthritis: Validated Staging and Scoring. J. Bone Jt. Surg. Am..

[6] Chu C.R., Millis M.B., Olson S.A. (2014). Osteoarthritis: From Palliation to Prevention: AOA Critical Issues. J. Bone Jt. Surg. Am..

[7] Felson D.T. (2013). Osteoarthritis as a Disease of Mechanics. Osteoarthr. Cartil..

[8] Mobasheri A., Rayman M.P., Gualillo O., Sellam J., van der Kraan P., Fearon U. (2017). The Role of Metabolism in the Pathogenesis of Osteoarthritis. Nat. Rev. Rheumatol..

[9] Roemer F.W., Crema M.D., Trattnig S., Guermazi A. (2011). Advances in Imaging of Osteoarthritis and Cartilage. Radiology.

[10] Kraus V.B., Burnett B., Coindreau J., Cottrell S., Eyre D., Gendreau M., Gardiner J., Garnero P., Hardin J., Henrotin Y. (2011). Application of Biomarkers in the Development of Drugs Intended for the Treatment of Osteoarthritis. Osteoarthr. Cartil..

[11] Bauer D.C., Hunter D.J., Abramson S.B., Attur M., Corr M., Felson D., Heinegård D., Jordan J.M., Kepler T.B., Lane N.E. (2006). Classification of Osteoarthritis Biomarkers: A Proposed Approach. Osteoarthr. Cartil..

[12] Tiulpin A., Thevenot J., Rahtu E., Lehenkari P., Saarakkala S. (2018). Automatic Knee Osteoarthritis Diagnosis from Plain Radiographs: A Deep Learning-Based Approach. Sci. Rep..

[13] Bay-Jensen A.C., Engstrom A., Sharma N., Karsdal M.A. (2020). Blood and Urinary Collagen Markers in Osteoarthritis: Markers of Tissue Turnover and Disease Activity. Osteoarthr. Cartil..

[14] Kolhe R., Hunter M., Liu S., Jadeja R.N., Pundkar C., Mondal A.K., Mendhe B., Drewry M., Rojiani M.V., Liu Y. (2017). Gender-Specific Differential Expression of Exosomal miRNA in Synovial Fluid of Patients with Osteoarthritis. Sci. Rep..

[15] Demehri S., Hafezi-Nejad N., Carrino J.A. (2015). Conventional and Novel Imaging Modalities in Osteoarthritis: Current State of the Evidence. Curr. Opin. Rheumatol..

[16] Saarakkala S., Waris P., Waris V., Tarkiainen I., Karvanen E., Aarnio J., Koski J.M. (2012). Diagnostic Performance of Knee Ultrasonography for Detecting Degenerative Changes of Articular Cartilage. Osteoarthr. Cartil..

[17] Alizai H., Walter W., Khodarahmi I., Burke C.J. (2019). Cartilage Imaging in Osteoarthritis. Semin. Musculoskelet. Radiol..

[18] Mahum R., Rehman S.U., Meraj T., Rauf H.T., Irtaza A., El-Sherbeeny A.M., El-Meligy M.A. (2021). A Novel Hybrid Approach Based on Deep CNN to Detect Knee Osteoarthritis. Sensors.

[19] Guida C., Zhang M., Shan J. (2023). Improving Knee Osteoarthritis Classification Using Multimodal Intermediate Fusion of X-ray, MRI, and Clinical Information. Neural Comput. Appl..

[20] Xuan A., Chen H., Chen T., Li J., Lu S., Fan T., Zeng D., Wen Z., Ma J., Hunter D. (2023). The Application of Machine Learning in Early Diagnosis of Osteoarthritis: A Narrative Review. Ther. Adv. Musculoskelet. Dis..

[21] Hao H.Q., Zhang J.F., He Q.Q., Wang Z. (2019). Cartilage Oligomeric Matrix Protein, C-terminal Cross-linking Telopeptide of Type II Collagen, and Matrix Metalloproteinase-3 as Biomarkers for Knee and Hip Osteoarthritis Diagnosis: A Systematic Review and Meta-analysis. Osteoarthr. Cartil..

[22] Lorenzo P., Aspberg A., Saxne T., Onnerfjord P. (2017). Quantification of Cartilage Oligomeric Matrix Protein (COMP) and a COMP Neoepitope in Synovial Fluid of Patients with Different Joint Disorders by Novel Automated Assays. Osteoarthr. Cartil..

[23] Styrkarsdottir U., Lund S.H., Saevarsdottir S., Magnusson M.I., Gunnarsdottir K., Norddahl G.L., Frigge M.L., Ivarsdottir E.V., Bjornsdottir G., Ingvarsson T. (2023). Cartilage Acidic Protein 1 in Plasma Associates with Prevalent Osteoarthritis and Predicts Future Risk as Well as Progression to Joint Replacements:Results from the UK Biobank Resource. Arthritis Rheumatol..

[24] Szilagyi I., Torsney K.M., Karsdal M.A., Bay-Jensen A.C., Welting T.J.M. (2023). Plasma Proteomics Identifies CRTAC1 as a Biomarker for Osteoarthritis Severity and Progression. Rheumatology.

[25] Joseph G.B., Nevitt M.C., McCulloch C.E., Neumann J., Lynch J.A., Heilmeier U., Lane N.E., Link T.M. (2018). Associations Between Molecular Biomarkers and MR-based Cartilage Composition and Knee Joint Morphology: Data from the Osteoarthritis Initiative. Osteoarthr. Cartil..

[26] Vilím V., Vytášek R., Olejarzová M., Macháček S., Gatterová J., Procházka L., Kraus V.B., Pavelka K. (2001). Serum Cartilage Oligomeric Matrix Protein Reflects the Presence of Clinically Diagnosed Synovitis in Patients with Knee Osteoarthritis. Osteoarthr. Cartil..

[27] Tseng S., Reddi A.H., Di Cesare P.E. (2009). Cartilage Oligomeric Matrix Protein (COMP): A Biomarker of Arthritis. Biomark. Insights.

[28] Garnero P., Piperno M., Gineyts E., Christgau S., Delmas P.D., Vignon E. (2001). Cross Sectional Evaluation of Biochemical Markers of Bone, Cartilage, and Synovial Tissue Metabolism in Patients with Knee Osteoarthritis: Relations with Disease Activity and Joint Damage. Ann. Rheum. Dis..

[29] Henrotin Y., Addison S., Kraus V., Deberg M. (2007). Type II collagen markers in osteoarthritis: What do they indicate?. Curr. Opin. Rheumatol..

[30] Rousseau J.C., Delmas P.D. (2007). Biological Markers in Osteoarthritis. Nat. Clin. Pract. Rheumatol..

[31] Scanzello C.R., Goldring S.R. (2012). The Role of Synovitis in Osteoarthritis Pathogenesis. Bone.

[32] Attur M., Krasnokutsky S., Statnikov A., Samuels J., Li Z., Friese O., Hellio Le Graverand M.P., Rybak L., Kraus V.B., Jordan J.M. (2015). Low-Grade Inflammation in Symptomatic Knee Osteoarthritis: Prognostic Value of Inflammatory Plasma Lipids and Peripheral Blood Leukocyte Biomarkers. Arthritis Rheumatol..

[33] Berenbaum F. (2013). Osteoarthritis as an Inflammatory Disease (Osteoarthritis Is Not Osteoarthrosis!). Osteoarthr. Cartil..

[34] Sokolove J., Lepus C.M. (2013). Role of Inflammation in the Pathogenesis of Osteoarthritis: Latest Findings and Interpretations. Ther. Adv. Musculoskelet. Dis..

[35] Robinson W.H., Lindstrom T.M., Cheung R.K., Sokolove J. (2013). Mechanistic Biomarkers for Clinical Decision Making in Rheumatic Diseases. Nat. Rev. Rheumatol..

[36] Ortona E., Pagano M.T., Capossela L., Malorni W., Gagliardi M.C. (2024). The Role of Sex in Osteoarthritis and Biomechanical Factors. Int. J. Mol. Sci..

[37] Geyer M., Schönfeld C. (2018). Novel Insights into the Pathogenesis of Osteoarthritis. Curr. Rheumatol. Rev..

[38] Liu F., Ding W., Fang Y., Yang J., Zhang Y., Ding C., Huang B., Wang K. (2024). Inflammatory Molecular Cluster in Knee Osteoarthritis Derived from Transcriptome and Machine Learning Analysis. Int. J. Mol. Sci..

[39] Felson D.T., Anderson J.J., Naimark A., Walker A.M., Meenan R.F. (1988). Obesity and Knee Osteoarthritis. The Framingham Study. Ann. Intern. Med..

[40] Sellam J., Berenbaum F. (2010). The Role of Synovitis in Pathophysiology and Clinical Symptoms of Osteoarthritis. Nat. Rev. Rheumatol..

[41] Wojdasiewicz P., Poniatowski Ł.A., Szukiewicz D. (2014). The Role of Inflammatory and Anti-inflammatory Cytokines in the Pathogenesis of Osteoarthritis. Mediat. Inflamm..

[42] Kapoor M., Martel-Pelletier J., Lajeunesse D., Pelletier J.P., Fahmi H. (2011). Role of Proinflammatory Cytokines in the Pathophysiology of Osteoarthritis. Nat. Rev. Rheumatol..

[43] Goldring M.B., Otero M. (2011). Inflammation in Osteoarthritis. Curr. Opin. Rheumatol..

[44] Stannus O., Jones G., Cicuttini F., Parameswaran V., Quinn S., Burgess J., Ding C. (2010). Circulating Levels of IL-6 and TNF-α Are Associated with Knee Radiographic Osteoarthritis and Knee Cartilage Loss in Older Adults. Osteoarthr. Cartil..

[45] Livshits G., Zhai G., Hart D.J., Kato B.S., Wang H., Williams F.M., Spector T.D. (2009). Interleukin-6 Is a Significant Predictor of Radiographic Knee Osteoarthritis: The Chingford Study. Arthritis Rheum..

[46] Schett G., Elewaut D., McInnes I.B., Dayer J.M., Neurath M.F. (2013). How Cytokine Networks Fuel Inflammation: Toward a Cytokine-Based Disease Taxonomy. Nat. Med..

[47] Hunter D.J., Felson D.T. (2006). Osteoarthritis. BMJ.

[48] Pearle A.D., Scanzello C.R., George S., Mandl L.A., DiCarlo E.F., Peterson M., Sculco T.P., Crow M.K. (2007). Elevated High-Sensitivity C-Reactive Protein Levels Are Associated with Local Inflammatory Findings in Patients with Osteoarthritis. Osteoarthr. Cartil..

[49] Spector T.D., Hart D.J., Nandra D., Doyle D.V., Mackillop N., Gallimore J.R., Pepys M.B. (1997). Low-Level Increases in Serum C-Reactive Protein Are Present in Early Osteoarthritis and Increase with Disease Progression. Arthritis Rheum..

[50] Punzi L., Ramonda R., Sfriso P. (2004). Erosive Osteoarthritis. Best Pract. Res. Clin. Rheumatol..

[51] Sun A.R., Wu X., Liu B., Chen Y., Armitage C.W., Kress H., Wang Z., Tian P., Crawford R., Prasadam I. (2015). Associations Among Inflammatory Biomarkers in the Circulating, Plasmatic, Salivary and Intraluminal Anatomical Compartments in Apparently Healthy Young Adults. PLoS ONE.

[52] Brzustewicz E., Bryl E. (2015). The role of cytokines in the pathogenesis of rheumatoid arthritis—Practical and potential application of cytokines as biomarkers and targets of personalized therapy. Cytokine.

[53] Boffa A., Merli G., Andriolo L., Lattermann C., Salzmann G.M., Filardo G. (2021). Synovial Fluid Biomarkers in Knee Osteoarthritis: A Systematic Review and Meta-Analysis. J. Clin. Med..

[54] Struglics A., Larsson S., Pramhed J., Frobell R., Sward P., Roemer F., Lohmander S., Kraus V. (2024). Osteoarthritis Biomarkers: Year in Review. Osteoarthr. Cartil..

[55] Jayadev C., Hulme C., Snelling S., Mohan R., Taylor P., Price A., Garrett A., Glyn-Jones S., Carr A., Arden N.K. (2012). The Influence of Preanalytical Variables on Soluble CD40 Ligand Levels in Patients with Knee Osteoarthritis. Int. J. Rheum. Dis..

[56] Valdes A.M., Meulenbelt I., Gardiner J., Bay-Jensen A.C., Karsdal M., Spector T.D., Slagboom P.E., Menni C., Christiansen C., van Meurs J.B. (2014). Large Scale Meta-Analysis of Urinary C-Telopeptide of Type II Collagen (uCTX-II) as a Biomarker of Osteoarthritis and Joint Diseases. Osteoarthr. Cartil..

[57] Eckstein F., Le Graverand M.P. (2015). Plain radiography or magnetic resonance imaging (MRI): Which is better in assessing outcome in clinical trials of disease-modifying osteoarthritis drugs? Summary of a debate held at the World Congress of Osteoarthritis 2014. Semin. Arthritis Rheum..

[58] Mosher T.J., Walker E.A., Petscavage-Thomas J., Guermazi A. (2013). Osteoarthritis Year 2013: Imaging. Osteoarthr. Cartil..

[59] Baumgartner T.R., Campos D.F., Hafezi-Nejad N., Demehri S. (2022). Advanced Imaging of Osteoarthritis: Radiography, CT, and Dual Energy CT. Radiol. Clin. N. Am..

[60] Wirth W., Maschek S., Roemer F.W., Eckstein F. (2017). Layer-Specific Analysis of Femorotibial Cartilage T2 Relaxation Time in Knees with and without Early Osteoarthritis. Osteoarthr. Cartil..

[61] Liebl H., Joseph G., Nevitt M.C., Singh N., Heilmeier U., Subburaj K., Jungmann P.M., Sharma L., Liu F., Wolski K. (2015). Early T2 Changes Predict Onset of Radiographic Knee Osteoarthritis: Data from the Osteoarthritis Initiative. Ann. Rheum. Dis..

[62] MacKay J.W., Low S.B., Smith T.O., Toms A.P., McCaskie A.W., Gilbert F.J. (2018). Systematic Review and Meta-Analysis of the Reliability and Discriminative Validity of Cartilage Compositional MRI in Knee Osteoarthritis. Osteoarthr. Cartil..

[63] van Tiel J., Bron E.E., Tiderius C.J., Bos P.K., Reijman M., Klein S., Verhaar J.A., Krestin G.P., Weinans H., Kotek G. (2013). Reproducibility of 3D Delayed Gadolinium Enhanced MRI of Cartilage (dGEMRIC) of the Knee at 3T in Patients with Early Stage Osteoarthritis. Osteoarthr. Cartil..

[64] Horga L.M., Hirschmann A.C., Henckel J., Fotiadou A., Di Laura A., Torlasco C., D’Silva A., Sharma S., Moon J.C., Hart A.J. (2020). Prevalence of Abnormal Findings in 230 Knees of Asymptomatic Adults Using 3.0 T MRI. Skelet. Radiol..

[65] Kijowski R., Blankenbaker D.G., Klaers J.L., Shinki K., De Smet A.A., Block W.F. (2009). Vastly undersampled isotropic projection steady-state free precession imaging of the knee: Diagnostic performance compared with conventional MR. Radiology.

[66] Cao P., Wu Y., Liang Y., Tang J., Wang Y., Wu Y., Liu X., Wang J., Wu J., Wu Y. (2024). Ultrasound Imaging in the Diagnosis and Assessment of Knee Osteoarthritis: A Systematic Review and Meta-Analysis. J. Ultrasound Med..

[67] Okano T., Filippucci E., Di Carlo M., Draghessi A., Carotti M., Salaffi F., Smerilli G., Di Matteo A., Cipolletta E., Terslev L. (2021). Ultrasonographic Evaluation of Joint Damage in Knee Osteoarthritis: Feature-Specific Comparisons with Conventional Radiography. Rheumatology.

[68] Ishibashi K., Sasaki E., Ota S., Chiba D., Yamamoto Y., Tsuda E., Yoshikuni S., Ihara K., Ishibashi Y. (2020). Detection of synovitis in early knee osteoarthritis by MRI and serum biomarkers in Japanese general population. Sci. Rep..

[69] Thoenen J., MacKay J.W., Sandford H.J.C., Gold G.E., Kogan F. (2022). Imaging of Synovial Inflammation in Osteoarthritis, From the AJR Special Series on Inflammation. AJR Am. J. Roentgenol..

[70] Peuna A., Hekkala J., Haapea M., Podlipská J., Guermazi A., Saarakkala S., Nieminen M.T., Lammentausta E. (2024). Complexity of Knee Osteoarthritis Pathogenesis: A Comprehensive Review of Clinical, Molecular, and Imaging Insights with a Call for Interdisciplinary Research. Arthritis Res. Ther..

[71] Riecke B.F., Christensen R., Torp-Pedersen S., Boesen M., Gudbergsen H., Bliddal H. (2014). An Ultrasound Score for Knee Osteoarthritis: A Cross-Sectional and Longitudinal Study. Osteoarthr. Cartil..

[72] Hall M., Doherty S., Courtney P., Latief K., Zhang W., Doherty M. (2014). Synovial Pathology Detected on Ultrasound Correlates with the Severity of Radiographic Knee Osteoarthritis More Than with Symptoms. Osteoarthr. Cartil..

[73] Wu P.T., Shao C.J., Wu K.C., Wu T.T., Chern T.C., Kuo L.C., Jou I.M. (2012). Pain in Patients with Equal Radiographic Grades of Osteoarthritis in Both Knees: The Value of Gray Scale Ultrasound. Osteoarthr. Cartil..

[74] Omoumi P., Babel H., Jolles B.M., Favre J. (2022). Imaging of Lower Limb Cartilage. Diagn. Interv. Imaging.

[75] Mallinson P.I., Coupal T.M., McLaughlin P.D., Nicolaou S., Munk P.L., Ouellette H.A. (2016). Dual-Energy CT for the Musculoskeletal System. Radiology.

[76] Heim B., Krismer F., De Marzi R., Seppi K. (2017). Magnetic resonance imaging for the diagnosis of Parkinson’s disease. J. Neural Transm..

[77] Fritz J., Guggenberger R., Del Grande F. (2021). Rapid Musculoskeletal MRI in 2021: Clinical Application of Advanced Accelerated Techniques. AJR Am. J. Roentgenol..

[78] Bowes M.A., Lohmander L.S., Wolstenholme C.B., Vincent G.R., Conaghan P.G., Frobell R.B. (2019). Marked and Rapid Change of Bone Shape in Acutely ACL Injured Knees: An Exploratory Analysis of the Kanon Study. Osteoarthr. Cartil..

[79] Hirvasniemi J., Klein S., Niessen W.J., Oei E.H., Bierma-Zeinstra S.M., Weinans H., Zadpoor A.A. (2022). MRI-Based Radiomic Features of the Tibial Bone Can Discriminate Between Knees Without and With Osteoarthritis: Data from the Osteoarthritis Initiative. J. Orthop. Res..

[80] Morales J., Rajaraman A., Antani S., Thoma G., Folio L., Burks G., Folio L. (2024). Radiomics-Based Detection of Osteoarthritis-Associated Changes in Trabecular Bone Texture Using Machine Learning. Diagnostics.

[81] Halilaj E., Le Y., Hicks J.L., Hastie T.J., Delp S.L. (2018). Modeling and Predicting Osteoarthritis Progression: Data from the Osteoarthritis Initiative. Osteoarthr. Cartil..

[82] Jamshidi A., Pelletier J.P., Martel-Pelletier J. (2019). Machine-Learning-Based Patient-Specific Prediction Models for Knee Osteoarthritis. Nat. Rev. Rheumatol..

[83] Jiang T., Lau S.H., Zhang J., Chan L.C., Wang W., Chan P.K., Cai J., Wen C. (2024). Radiomics signature of osteoarthritis: Current status and perspective. J. Orthop. Translat..

[84] Collins G.S., Reitsma J.B., Altman D.G., Moons K.G. (2015). Transparent Reporting of a Multivariable Prediction Model for Individual Prognosis or Diagnosis (TRIPOD): The TRIPOD Statement. Ann. Intern. Med..

[85] Schwendicke F., Golla T., Dreher M., Krois J. (2019). Convolutional Neural Networks for Dental Image Diagnostics: A Scoping Review. J. Dent..

[86] Paderno A., Holsinger F.C., Piazza C. (2021). Videomics: Bringing Deep Learning to Diagnostic Endoscopy. Curr. Opin. Otolaryngol. Head Neck Surg..

[87] van der Haar P., Oei E.H., Runhaar J., Bierma-Zeinstra S.M., Klein S., Weinans H. (2024). Multimodal Machine Learning-Based Knee Osteoarthritis Progression Prediction: Results from the OAI Study. J. Orthop. Res..

[88] Pedoia V., Lee J., Norman B., Link T.M., Majumdar S. (2019). Diagnosing osteoarthritis from T2 maps using deep learning: An analysis of the entire Osteoarthritis Initiative baseline cohort. Osteoarthr. Cartil..

[89] Roemer F.W., Eckstein F., Hayashi D., Guermazi A. (2014). The Role of Imaging in Osteoarthritis. Best Pract. Res. Clin. Rheumatol..

[90] Krustev E., Rioux D., McDougall J.J. (2021). A Protocol for the Use of DMM/PTX-Induced Mouse Models of Osteoarthritis and Rheumatoid Arthritis. Curr. Protoc..

[91] Haubruck P., Pinto M., Moradi B., Little C.B., Camus T. (2024). Monocyte Recruitment and Activated Macrophage Functions in a Murine Model of Knee Osteoarthritis. Int. J. Mol. Sci..

[92] Almhdie-Imjabbar A., Toumi H., Harrar K., Mokrani K., Lespessailles E. (2024). Machine Learning Approaches for Osteoporosis Screening Using Vertebral Bone Quality and Bone Mineral Density from CT Images. Diagnostics.

[93] Rastegar S., Sepehri M.M., Baradaran Mahdavi S., Javadi S., Kan F., Ghaffari A., Ershadi S., Ghorbani M., Hosseinzadeh M., Jamshidi A. (2024). Multimodal Machine Learning for Modeling Knee Osteoarthritis Progression Using Radiographic, Metabolic, and Clinical Data. Arthritis Res. Ther..

[94] Li M.D., Chang K., Bearce B., Chang C.Y., Huang A.J., Campbell J.P., Brown J.M., Singh P., Hoebel K.V., Erdoğmuş D. (2020). Siamese Neural Networks for Continuous Disease Severity Evaluation and Change Detection in Medical Imaging. NPJ Digit. Med..

[95] Guermazi A., Roemer F.W., Hayashi D., Crema M.D., Niu J., Zhang Y., Marra M.D., Katur A., Lynch J.A., El-Khoury G.Y. (2011). Assessment of synovitis with contrast-enhanced MRI using a whole-joint semiquantitative scoring system in people with, or at high risk of, knee osteoarthritis: The MOST study. Ann. Rheum. Dis..

[96] Chang G.H., Felson D.T., Qiu S., Guermazi A., Capellini T.D., Kolachalama V.B. (2024). Combining Multimodal Imaging to Improve Diagnosis and Prognosis in Knee Osteoarthritis. Osteoarthr. Cartil..

[97] Xu Y., Ding K., Wang Y., Zhang Y., Yang J., Li Y., Wang K., Ding C., Huang B. (2024). Multimodal Machine Learning Model Based on FLAG and 3D-CNN for Diagnosis and Prognosis Prediction in Knee Osteoarthritis. Arthritis Res. Ther..

[98] Deveza L.A., Nelson A.E., Loeser R.F. (2019). Phenotypes and Biomarkers in Osteoarthritis: Where Are We Now?. Curr. Opin. Rheumatol..

[99] Lazzarini N., Runhaar J., Bay-Jensen A.C., Thudium C.S., Bierma-Zeinstra S.M., Henrotin Y., Mobasheri A. (2017). A Machine Learning Approach for the Identification of New Biomarkers for Knee Osteoarthritis Development in Overweight and Obese Women. Osteoarthr. Cartil..

[100] Wu J., Li Y., Li Y., Wang Q., Han Y., Wang Y., Wang Y., Zhang Y., Ding C., Huang B. (2024). Correlation Analysis of Serum Biomarkers with Imaging Features and Knee Osteoarthritis Phenotypes: Data from the Osteoarthritis Initiative. J. Clin. Med..

[101] Conaghan P.G., Katz N., Hunter D.J., Guermazi A., Hochberg M.C., Somberg K., Clive J., Knight C., Johnson M., Zhao L. (2024). Exploring a novel outcome measure of symptom progression in knee osteoarthritis utilizing a large randomized trial. Osteoarthr. Cartil..

[102] Huang C., Song P., Huang M., Lin Z., Liu B., Huang Y., Wang Y., Ding C., Wang K. (2024). Machine Learning Uncovers Novel Markers from Single-Cell RNA-Seq Data for Knee Osteoarthritis. Int. J. Mol. Sci..

[103] Guan Z., Lin Z., Huang M., Huang C., Song P., Wang Y., Ding C., Huang Y., Wang K. (2024). Clustering Analysis of Knee Osteoarthritis Patients Based on Synovial Fluid Multi-Omics Data. Arthritis Res. Ther..

[104] Thudium C.S., Karsdal M.A., Bay-Jensen A.C. (2024). Prediction of Knee Osteoarthritis Progression Using Epidemiological and Molecular Profiles: A Machine Learning Approach. Arthritis Res. Ther..

[105] Sun H., Zhang Y., Wang Y., Liu J., Ding C., Huang B., Wang K. (2024). Integration of Multi-Omics and Clinical Data for Prediction of Knee Osteoarthritis Progression. J. Clin. Med..

[106] Bihlet A.R., Byrjalsen I., Bay-Jensen A.C., Andersen J.R., Christiansen C., Riis B.J., Karsdal M.A. (2019). Associations Between Biomarkers of Bone and Cartilage Turnover, Gender, Pain Categories and Radiographic Severity in Knee Osteoarthritis. Arthritis Res. Ther..

[107] Nelson A.E., Fang F., Arbeeva L., Cleveland R.J., Schwartz T.A., Callahan L.F., Marron J.S., Loeser R.F. (2019). A Machine Learning Approach to Knee Osteoarthritis Phenotyping: Data from the FNIH Biomarkers Consortium. Osteoarthr. Cartil..

[108] Driban J.B., Sitler M.R., Barbe M.F., Balasubramanian E. (2014). Is Pain in One Knee Associated with Isometric Muscle Strength in the Contralateral Limb? Data from the Osteoarthritis Initiative. Am. J. Phys. Med. Rehabil..

[109] Øiestad B.E., Juhl C.B., Eitzen I., Thorlund J.B. (2015). Knee Extensor Muscle Weakness Is a Risk Factor for Development of Knee Osteoarthritis. A Systematic Review and Meta-Analysis. Osteoarthr. Cartil..

[110] Bastick A.N., Runhaar J., Belo J.N., Bierma-Zeinstra S.M. (2015). Prognostic Factors for Progression of Clinical Osteoarthritis of the Knee: A Systematic Review of Observational Studies. Arthritis Res. Ther..

[111] Mehta B., Goodman S., DiCarlo E., Jannat-Khah D., Gibbons J.A.B., Otero M., Donlin L., Pannellini T., Robinson W.H., Sculco P. (2023). Machine learning identification of thresholds to discriminate osteoarthritis and rheumatoid arthritis synovial inflammation. Arthritis Res. Ther..

[112] Schiratti J.B., Allassonnière S., Colliot O., Durrleman S. (2017). A Bayesian Mixed-Effects Model to Learn Trajectories of Changes from Repeated Manifold-Valued Observations. J. Mach. Learn. Res..

[113] Joseph G.B., McCulloch C.E., Nevitt M.C., Neumann J., Gersing A.S., Kretzschmar M., Schwaiger B.J., Lynch J.A., Heilmeier U., Lane N.E. (2018). Tool for Osteoarthritis Risk Prediction (TOARP) Over 8 Years Using Baseline Clinical Data, X-Ray, and MRI: Data from the Osteoarthritis Initiative. J. Magn. Reson. Imaging.

[114] Wang W., Yan X., Wei J., Wang Y., Zhang Y., Ding C., Huang B., Wang K. (2024). Multi-View Learning for Knee Osteoarthritis Severity Classification Using Radiomic Features. J. Clin. Med..

[115] Bhatt S., Cohon A., Rose J., Majerczyk N., Cozzi B., Crenshaw D., Myers G. (2021). Interpretable Machine Learning Models for Clinical Decision-Making in a High-Need, Value-Based Primary Care Setting. NEJM Catal..

[116] Tiulpin A., Klein S., Bierma-Zeinstra S.M.A., Thevenot J., Rahtu E., van Meurs J., Oei E.H.G., Saarakkala S. (2019). Multimodal Machine Learning-based Knee Osteoarthritis Progression Prediction from Plain Radiographs and Clinical Data. Sci. Rep..

[117] Almhdie-Imjabbar A., Toumi H., Lespessailles E. (2024). Machine Learning-Based Classification of Osteoporotic Vertebral Fractures Using Radiomics Features from CT Images. Diagnostics.

[118] D’Antoni F., Russo F., Ambrosio L., Borthakur A., Scafoglieri A., Beckwée D., Cattrysse E. (2024). The Diagnostic Accuracy of Conventional MRI and MRI-Based GSI Quantitative Histogram Analysis in Distinguishing Low-Grade from High-Grade Chondrosarcoma. Diagnostics.

[119] Perry D.C., Arch B., Appelbe D., Francis P., Craven J., Monsell F.P., Williamson P., Knight M. (2022). The British Orthopaedic Surgery Surveillance Study: Slipped Capital Femoral Epiphysis: The Epidemiology and Two-Year Outcomes from a Prospective UK Cohort. Bone Jt. J..

[120] Konin G.P., Walz D.M. (2010). Lumbosacral Transitional Vertebrae: Classification, Imaging Findings, and Clinical Relevance. AJNR Am. J. Neuroradiol..

[121] Knol M.J., Le Cessie S., Algra A., Vandenbroucke J.P., Groenwold R.H. (2012). Overestimation of Risk Ratios by Odds Ratios in Trials and Cohort Studies: Alternatives to Logistic Regression. CMAJ.

[122] Hosmer D.W., Lemeshow S., May S. (2009). Applied Survival Analysis: Regression Modeling of Time-to-Event Data.

[123] Altman D.G., Bland J.M. (1994). Diagnostic Tests 2: Predictive Values. BMJ.

[124] Sackett D.L., Haynes R.B. (2002). The Architecture of Diagnostic Research. BMJ.

[125] Rembold C.M. (1998). Number Needed to Screen: Development of a Statistic for Disease Screening. BMJ.

[126] Whiting P.F., Rutjes A.W., Westwood M.E., Mallett S., Deeks J.J., Reitsma J.B., Leeflang M.M., Sterne J.A., Bossuyt P.M., QUADAS-2 Group (2011). QUADAS-2: A Revised Tool for the Quality Assessment of Diagnostic Accuracy Studies. Ann. Intern. Med..

[127] Schünemann H.J., Oxman A.D., Brożek J., Glasziou P., Jaeschke R., Vist G.E., Williams J.W., Kunz R., Craig J., Montori V.M. (2008). Grading Quality of Evidence and Strength of Recommendations for Diagnostic Tests and Strategies. BMJ.

[128] Deeks J.J., Altman D.G. (2004). Diagnostic Tests 4: Likelihood Ratios. BMJ.

[129] Zhang W., Doherty M., Leeb B.F., Alekseeva L., Arden N.K., Bijlsma J.W., Dinçer F., Dziedzic K., Häuselmann H.J., Herrero-Beaumont G. (2007). EULAR evidence based recommendations for the management of hand osteoarthritis: Report of a Task Force of the EULAR Standing Committee for International Clinical Studies Including Therapeutics (ESCISIT). Ann. Rheum. Dis..

[130] Zikria B., Rinaldi J., Guermazi A., Roemer F.W., Demehri S., Murakami A.M. (2024). The Role of Imaging in Osteoarthritis Diagnosis and Management. Life.

[131] Hayashi D., Roemer F.W., Guermazi A. (2018). Imaging of osteoarthritis-recent research developments and future perspective. Br. J. Radiol..

[132] Kumar R., Gowda C., Sekhar T.C., Vaja S., Hage T., Sporn K., Waisberg E., Ong J., Zaman N., Tavakkoli A. (2025). Advancements in Machine Learning for Precision Diagnostics and Surgical Interventions in Interconnected Musculoskeletal and Visual Systems. J. Clin. Med..

[133] Kwolek Ł., Kruczyński J., Fryzowicz A., Jurkiewicz A., Czernicki K., Czwojdziński A., Gregori M., Błażkiewicz M., Szulc A., Klebaniuk J. (2024). Radiographic Parameters of Knee Osteoarthritis and Patient-Reported Outcome Measures: A Cross-Sectional Study of a Polish Population. J. Clin. Med..

[134] Waszczykowski M., Fabiś-Strobin A., Bednarski I., Lesiak A., Narbutt J., Fabiś J. (2020). Serum Biomarkers of Inflammation and Turnover of Joint Cartilage Can Help Differentiate Psoriatic Arthritis (PsA) Patients from Osteoarthritis (OA) Patients. Diagnostics.

[135] Puts S., Njemini R., Bilterys T., Lefeber N., Scheerlinck T., Nijs J., Beckwée D., Bautmans I. (2024). Linking Intra-Articular Inflammatory Biomarkers with Peripheral and Central Sensitization in Late-Stage Knee Osteoarthritis Pain: A Pilot Study. J. Clin. Med..

[136] Yu S.P., Deveza L.A., Kraus V.B., Karsdal M., Bay-Jensen A.C., Collins J.E., Guermazi A., Roemer F.W., Ladel C., Bhagavath V. (2024). Association of Biochemical Markers with Bone Marrow Lesion Changes on Imaging—Data from the Foundation for the National Institutes of Health Osteoarthritis Biomarkers Consortium. Arthritis Res. Ther..

[137] Panizzi L., Vignes M., Dittmer K.E., Waterland M.R., Rogers C.W., Sano H., McIlwraith C.W., Riley C.B. (2024). Infrared Spectroscopy of Synovial Fluid Shows Accuracy as an Early Biomarker in an Equine Model of Traumatic Osteoarthritis. Animals.

[138] O’Sullivan O., Ladlow P., Steiner K., Kuyser D., Ali O., Stocks J., Valdes A.M., Bennett A.N., Kluzek S. (2023). Knee MRI Biomarkers Associated with Structural, Functional and Symptomatic Changes at Least a Year from ACL Injury—A Systematic Review. Osteoarthr. Cartil. Open.

[139] Gulshan V., Peng L., Coram M., Stumpe M.C., Wu D., Narayanaswamy A., Venugopalan S., Widner K., Madams T., Cuadros J. (2016). Development and Validation of a Deep Learning Algorithm for Detection of Diabetic Retinopathy in Retinal Fundus Photographs. JAMA.

[140] Han J., Han Y., Song Y., Kim J., Lee H., Lee S., Kim S.J., Nam Y., Park S., Lee J. (2024). Deep Learning-Based Outcome Prediction Using Pre- and Post-Treatment MRI in Patients with Lumbar Disc Herniation. Diagnostics.

[141] Zoad R., Farooq M., Khera O., Tahir M., Malik S., Adhikari A., Osman H., Mostafa J.A., Elkafrawy P., Azad A. (2024). Transforming Healthcare Cybersecurity: A Prescription for Resilience. Cureus.

[142] Aarons G.A., Hurlburt M., Horwitz S.M. (2011). Advancing a Conceptual Model of Evidence-Based Practice Implementation in Public Service Sectors. Adm. Policy Ment. Health.

[143] Damschroder L.J., Aron D.C., Keith R.E., Kirsh S.R., Alexander J.A., Lowery J.C. (2009). Fostering Implementation of Health Services Research Findings into Practice: A Consolidated Framework for Advancing Implementation Science. Implement. Sci..

[144] Feldstein A.C., Glasgow R.E. (2008). A Practical, Robust Implementation and Sustainability Model (PRISM) for Integrating Research Findings into Practice. Jt. Comm. J. Qual. Patient Saf..

[145] Glasgow R.E., Vogt T.M., Boles S.M. (1999). Evaluating the Public Health Impact of Health Promotion Interventions: The RE-AIM Framework. Am. J. Public Health.

[146] Ericsson K.A. (2004). Deliberate Practice and the Acquisition and Maintenance of Expert Performance in Medicine and Related Domains. Acad. Med..

[147] Kneeland P.P., Fang M.C. (2024). Improving Diagnostic Reasoning to Improve Patient Safety. Med. Clin. North Am..

[148] Ericsson K.A., Prietula M.J., Cokely E.T. (2007). The Making of an Expert. Harv. Bus. Rev..

[149] Kumar R., Sporn K., Khanna A., Paladugu P., Gowda C., Ngo A., Jagadeesan R., Zaman N., Tavakkoli A. (2025). Integrating Radiogenomics and Machine Learning in Musculoskeletal Oncology Care. Diagnostics.

[150] Neumann P.J., Sanders G.D., Russell L.B., Siegel J.E., Ganiats T.G. (2017). Cost-Effectiveness in Health and Medicine.

[151] (2016). Medical Devices—Quality Management Systems—Requirements for Regulatory Purposes.

[152] Van Calster B., Wynants L., Verbeke G., Van Huffel S., Valentin L., Timmerman D. (2018). Reporting and Interpreting Decision Curve Analysis: A Guide for Investigators. Eur. Urol..

[153] Obuchowski N.A., Bullen J.A. (2018). Receiver operating characteristic (ROC) curves: Review of methods with applications in diagnostic medicine. Phys. Med. Biol..

[154] Moons K.G., Altman D.G., Reitsma J.B., Ioannidis J.P., Macaskill P., Steyerberg E.W., Vickers A.J., Ransohoff D.F., Collins G.S. (2015). Transparent Reporting of a Multivariable Prediction Model for Individual Prognosis or Diagnosis (TRIPOD): Explanation and Elaboration. Ann. Intern. Med..

[155] Welhaven H.D., Welfley A.H., June R.K. (2025). Osteoarthritis Year in Review 2024: Molecular Biomarkers of Osteoarthritis. Osteoarthr. Cartil..

[156] Huang M., Song P., Guan Z., Liu B., Huang Y., Wang Y., Ding C., Wang K. (2024). Single-Cell Transcriptomics Reveals Molecular Characteristics of Synovial Tissue in Knee Osteoarthritis. Int. J. Mol. Sci..

[157] Song P., Huang C., Liu B., Guan Z., Wang Y., Ding C., Huang Y., Wang K. (2024). Proteomic Analysis Identifies Potential Biomarkers for Knee Osteoarthritis Progression. J. Clin. Med..

[158] Lin Z., Huang M., Song P., Guan Z., Liu B., Huang Y., Wang Y., Ding C., Wang K. (2024). Metabolomic Profiling of Synovial Fluid in Knee Osteoarthritis: Identification of Potential Biomarkers. Metabolites.

[159] Zhang Y., Wang Y., Song P., Huang C., Liu B., Guan Z., Huang Y., Ding C., Wang K. (2024). Spatial Transcriptomics Reveals Cellular Heterogeneity in Osteoarthritic Synovial Tissue. Arthritis Rheumatol..

[160] Gu Y., Hu Y., Zhang H., Wang S., Xu K., Su J. (2023). Single-cell RNA sequencing in osteoarthritis. Cell Prolif..

[161] Bay-Jensen A.C., Thudium C.S., Mobasheri A. (2018). Development and use of biochemical markers in osteoarthritis: Current update. Curr. Opin. Rheumatol..

[162] Lotz M., Martel-Pelletier J., Christiansen C., Brandi M.L., Bruyère O., Chapurlat R., Collette J., Cooper C., Giacovelli G., Kanis J.A. (2013). Value of Biomarkers in Osteoarthritis: Current Status and Perspectives. Ann. Rheum. Dis..

[163] Xie Y., Wang Y., Zhao X., Zhang Y., Ding C., Huang B., Wang K. (2023). Radiomics Analysis of Cartilage and Subchondral Bone to Differentiate Knees Predisposed to Posttraumatic Osteoarthritis. Osteoarthr. Cartil..

[164] Yu K., Ying J., Zhao T., Lei L., Zhong L., Hu J., Zhou J.W., Huang C., Zhang X. (2023). Prediction model for knee osteoarthritis using magnetic resonance-based radiomic features from the infrapatellar fat pad: Data from the osteoarthritis initiative. Quant. Imaging Med Surg..

[165] Li Y., Wang Y., Zhao X., Zhang Y., Ding C., Huang B., Wang K. (2023). Automatic Grading Model for Knee Osteoarthritis Using Plain Radiograph Radiomics. J. Clin. Med..

[166] Teoh Y.J., Lai K.W., Usman J., Goh S.L., Mohafez H., Hasikin K., Su K., Chu H.C., Wu X., Dhanalakshmi S. (2024). Exploring the Biomechanical and Biochemical Signatures of Knee Osteoarthritis: Towards Personalized Diagnostics and Targeted Interventions. Diagnostics.

[167] Kazemi M., Yu C., Mehrotra D.R., Ersland E.E., Zbyn S., Korna F., Staffa S.J., Engiles J.B., Li X., Schaer T.P. (2025). Raman Spectroscopic Probe Provides Optical Biomarkers of Cartilage Composition Predictive of Tissue Function. Osteoarthr. Cartil..

[168] Aicher W.K., Rolauffs B. (2014). The Spatial Organisation of Joint Surface Chondrocytes: Review of Its Potential Roles in Tissue Functioning, Disease and Early, Preclinical Diagnosis of Osteoarthritis. Ann. Rheum. Dis..

[169] Caravan P., Das B., Dumas S., Epstein F.H., Helm P.A., Jacques V., Koerner S., Kolodziej A., Shen L., Sun W.C. (2007). Collagen-Targeted MRI Contrast Agent for Molecular Imaging of Fibrosis. Angew. Chem. Int. Ed. Engl..

[170] Lee H., Park W., Lee J., Park J., Kim S., Park S., Lee J., Kim J., Han Y., Song Y. (2022). Photoacoustic Imaging for Non-Invasive Assessment of Knee Osteoarthritis: A Preliminary Study. Photoacoustics.

[171] Juras V., Szomolanyi P., Serfaty J.M., Chang G., Regatte R.R., Trattnig S. (2019). Sodium Imaging at Ultra-High Field MRI: A New Frontier in Musculoskeletal Imaging. NMR Biomed..

[172] Menendez M.I., Hettlich B., Wei L., Knopp M.V. (2018). Dynamic Contrast-Enhanced MRI in Murine Arthritis Models: Evaluation of Perfusion and Inflammation. J. Magn. Reson. Imaging.

[173] Subburaj K., Valentinitsch A., Dillon A.B., Pandit P., Li X., Link T.M., Vail T.P., Majumdar S. (2015). Quantitative Evaluation of Bone and Cartilage Changes Using Multispectral MR Imaging Following Bone Marrow Stimulation in a Porcine Model. J. Magn. Reson. Imaging.

[174] de Vries B.M., Zwezerijnen G.J.C., Burchell G.L., van Velden F.H.P., Menke-van der Houven van Oordt C.W., Boellaard R. (2023). Explainable artificial intelligence (XAI) in radiology and nuclear medicine: A literature review. Front. Med..

[175] Reyes M., Meier R., Pereira S., Silva C.A., Dahl A.L., Brox T., Maier-Hein L., Handels H., Reyes M., Shimizu A. (2020). On the Interpretability of Artificial Intelligence in Radiology: Challenges and Opportunities. Radiol. Artif. Intell..

[176] Zhang Y., Weng Y., Lund J. (2022). Applications of Explainable Artificial Intelligence in Diagnosis and Surgery. Diagnostics.

[177] Koh P.W., Nguyen T., Tang Y.S., Mussmann S., Pierson E., Kim B., Liang P. (2020). Concept Bottleneck Models. Proc. Mach. Learn. Res..

[178] Amann J., Blasimme A., Vayena E., Frey D., Madai V.I., Precise4Q Consortium (2020). Explainability for Artificial Intelligence in Healthcare: A Multidisciplinary Perspective. BMC Med. Inform. Decis. Mak..

[179] Rieke N., Hancox J., Li W., Milletari F., Roth H.R., Albarqouni S., Bakas S., Galtier M.N., Landman B.A., Maier-Hein K. (2020). The Future of Digital Health with Federated Learning. NPJ Digit. Med..

[180] Dayan I., Roth H.R., Zhong A., Harouni A., Gentili A., Abidin A.Z., Liu A., Costa A.B., Wood B.J., Tsai C.S. (2021). Federated Learning for Predicting Clinical Outcomes in Patients with COVID-19. Nat. Med..

[181] Kaissis G.A., Makowski M.R., Rückert D., Braren R.F. (2020). Secure, Privacy-Preserving and Federated Machine Learning in Medical Imaging. Nat. Mach. Intell..

[182] Sheller M.J., Edwards B., Reina G.A., Martin J., Pati S., Kotrotsou A., Milchenko M., Xu W., Marcus D., Colen R.R. (2020). Federated learning in medicine: Facilitating multi-institutional collaborations without sharing patient data. Sci. Rep..

[183] Pfitzner B., Steckhan N., Arnrich B. (2021). Federated Learning in a Medical Context: A Systematic Literature Review. ACM Trans. Internet Technol..

[184] Ng D., Lan X., Yao M.M., Chan W.P., Feng M. (2021). Federated Learning: A Collaborative Effort to Achieve Better Medical Imaging Models for Individual Sites That Have Small Labelled Datasets. Quant. Imaging Med. Surg..

